# Nerve Ultrasound as Helpful Tool in Polyneuropathies

**DOI:** 10.3390/diagnostics11020211

**Published:** 2021-01-31

**Authors:** Magdalena Kramer, Alexander Grimm, Natalie Winter, Marc Dörner, Kathrin Grundmann-Hauser, Jan-Hendrik Stahl, Julia Wittlinger, Josua Kegele, Cornelius Kronlage, Sophia Willikens

**Affiliations:** 1 Department of Neurology and Epileptology, Tübingen University Hospital and Hertie Institute for Clinical Brain Research, Eberhard Karls University Tübingen, 72076 Tübingen, Germany; magdalena.kramer@med.uni-tuebingen.de (M.K.); natalie.winter@med.uni-tuebingen.de (N.W.); marc.doerner@med.uni-tuebingen.de (M.D.); jan-hendrik.stahl@med.uni-tuebingen.de (J.-H.S.); julia.wittlinger@med.uni-tuebingen.de (J.W.); josua.kegele@med.uni-tuebingen.de (J.K.); cornelius.kronlage@med.uni-tuebingen.de (C.K.); sophia.willikens@med.uni-tuebingen.de (S.W.); 2 Department of Forensic Psychiatry, University Hospital of Psychiatry Zurich, University of Zurich, 8008 Zurich, Switzerland; 3 Institute of Medical Genetics and Applied Genomics, Tübingen University Hospital, Eberhard Karls University Tübingen, 72076 Tübingen, Germany; kathrin.grundmann@med.uni-tuebingen.de

**Keywords:** high-resolution nerve ultrasound, peripheral nerve imaging, demyelinating polyneuropathies, hereditary polyneuropathies

## Abstract

Background: Polyneuropathies (PNP) are a broad field of diseases affecting millions of people. While the symptoms presented are mostly similar, underlying causes are abundant. Thus, early identification of treatable causes is often difficult. Besides clinical data and basic laboratory findings, nerve conduction studies are crucial for etiological classification, yet limited. Besides Magnetic Resonance Imaging (MRI), high-resolution nerve ultrasound (HRUS) has become a noninvasive, fast, economic and available tool to help distinguish different types of nerve alterations in neuropathies. Methods: We aim to describe typical ultrasound findings in PNP and patterns of morphological changes in hereditary, immune-mediated, diabetic, metabolic and neurodegenerative PNP. Literature research was performed in PubMed using the terms ‘nerve ultrasound’, neuromuscular ultrasound, high-resolution nerve ultrasound, peripheral nerves, nerve enlargement, demyelinating, hereditary, polyneuropathies, hypertrophy’. Results: Plenty of studies over the past 20 years investigated the value of nerve ultrasound in different neuropathies. Next to nerve enlargement, patterns of nerve enlargement, echointensity, vascularization and elastography have been evaluated for diagnostic terms. Furthermore, different scores have been developed to distinguish different etiologies of PNP. Conclusions: Where morphological alterations of the nerves reflect underlying pathologies, early nerve ultrasound might enable a timely start of available treatment and also facilitate follow up of therapy success.

## 1. Introduction

Polyneuropathies are among the most frequent neurological disorders and cause significant morbidity and use of resources. Finding the cause of neuropathic deficits still remains a challenge in daily clinical routine. Although technical tools have improved and nerve conduction studies, skin and nerve biopsy, next generation sequencing, cerebrospinal fluid (CSF) analysis or antibody testing are established methods, the cause of PNP remains unclear in up to one third of patients. Imaging tools such as MRI of the roots and nerves could contribute to the diagnosis of immune-mediated neuropathies, hereditary transthyretin (TTR) associated systemic amyloidosis and others in the recent past [1,2,3]. However, their use is still limited to specialized centers. In contrast, ultrasound is a widely distributed tool, which is able to visualize nerves and roots with high resolution and accuracy. A growing number of studies has been published in the last decade concerning ultrasound findings in polyneuropathies [4,5]. The aim of this review is to give an overview and to propose an algorithm for examination of patients with suspected PNP including nerve ultrasound and nerve conduction studies.

## 2. Ultrasound Parameters in Neuropathies

**Figure 1:**
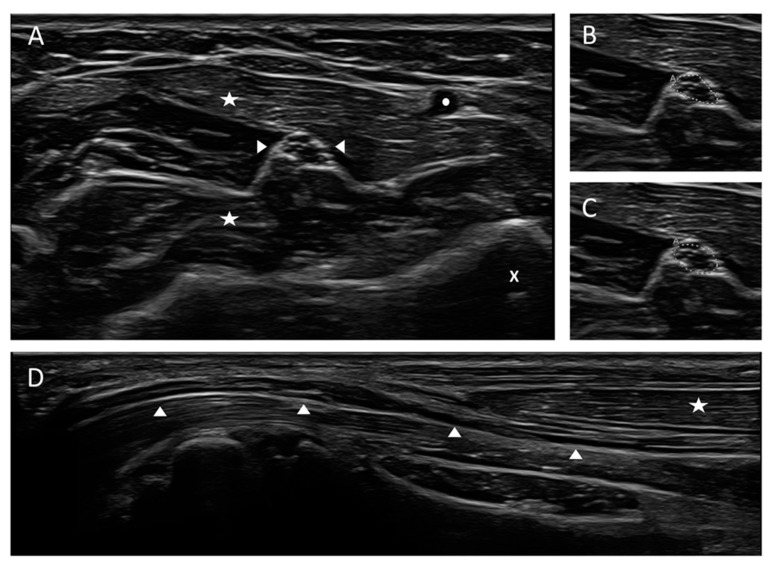
Cross-sectional and longitudinal view of a normal median nerve at forearm level. (**A**) Cross-sectional view of the median nerve. The nerve appearance is honey-comb-like with hypoechoic fascicles and hyperechoic epi- and perineurium. (**B**,**C**) Measurement techniques of the cross-sectional area (CSA) using the free-hand (**B**, upper picture) or ellipse function (**C**, lower picture) of the ultrasound device, CSA 5 mm^2^. (**D**) Longitudinal view of the median nerve in panoramic view. Left side of the picture: entrance into carpal tunnel. Arrow heads are marking the nerve, asterisks the superficial and flexor digitorum muscles, the circle the radial artery and the x the radial bone, “A” in pictures B and C means “area”.

**Figure 2:**
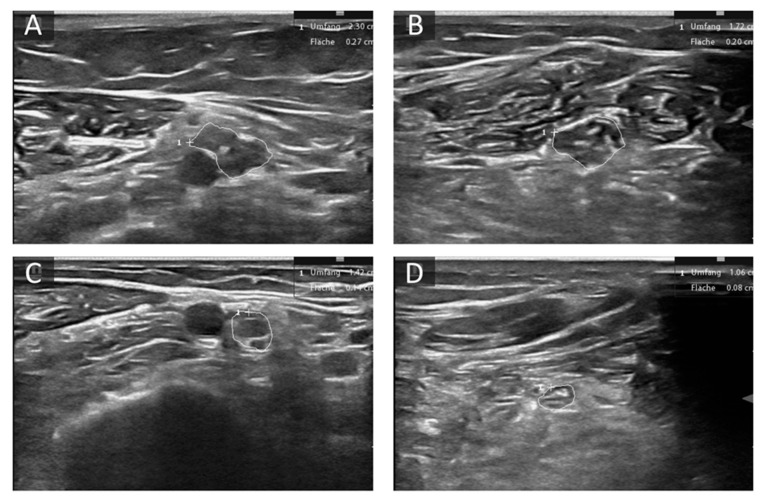
Examples of altered nerve morphology in hereditary neuropathies. (**A**,**B**) Cross-sectional view of the median nerve at the upper arm (**A**) and forearm (**B**) of a patient with Charcot Marie Tooth (CMT) 1A. CSA is enlarged (27 mm^2^ upper arm, 20 mm^2^ forearm) and the fascicles are hypoechoic. (**C**,**D**) Cross-sectional view of the median nerve at the upper arm (**C**) and forearm (**D**) of a patient with transthyretin related amyloidosis (ATTR). CSA is mildly enlarged at the upper arm (14 mm^2^) and normal at forearm level (8 mm^2^). The area enclosed by circles depicts CSA of the described nerve section, “+” shows curser position, “1” the count of measured areae.

**Figure 3:**
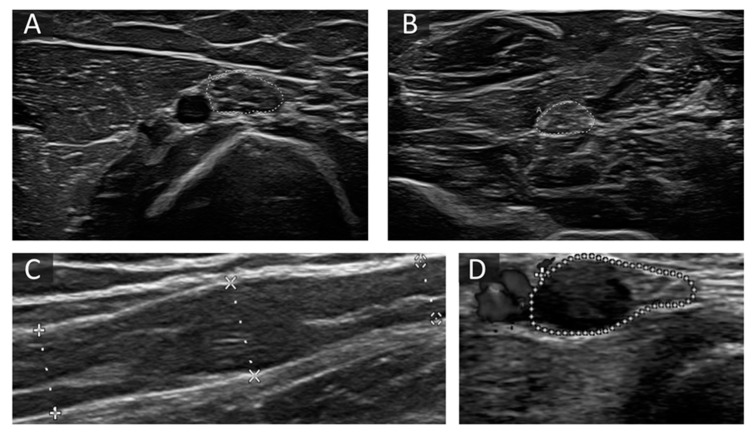
Examples of altered nerve morphology in immune-mediated neuropathies. (**A**,**B**) Cross-sectional view of the median nerve at the upper arm (30 mm^2^) (**A**) and forearm (17 mm^2^) (**B**) in a patient with chronic inflammatory demyelinating polyneuropathies (CIDP). CSA is predominantly enlarged at proximal sites, and fascicles are iso- and hyperechoic. (**C**) shows a long-axis view of a segmentally enlarged median nerve at the upper arm in a patient with Lewis-Sumner Syndrome (LSS). (**D**) The short-axis view shows an ulnar nerve at the forearm next to the ulnar artery with one very large fascicle with hypoechoic signal next to preserved fascicles in a patient with multifocal motor neuropathy (MMN). The area enclosed by the discontinuous points in picture A, B and D depict CSA, in picture C diameter of the described nerve section. “A” in pictures B and D means “area”.

Several aspects can be visualized and measured by ultrasound [6,7]. Before scanning the nerves, the examiner must be aware of the normal nerve appearance, especially in regard to nerve size, nerve and fascicle anatomy, echointensity, vascularity, elastography and surrounding tissue morphology (example is given in Figure 1). For differentiation of PNP the following parameters have been proven to be most useful: (1) The most simple feature is the enlargement of cross-sectional area (CSA), which can be seen either focally restricted (one short nerve segment), regionally (larger nerve segments) or diffusely (the whole nerve) within one nerve (unifocal), several nerves (multifocal) or all nerves and roots (generalized) (Figure 2). Further, nerve enlargement can exhibit certain predominance (e.g., proximal enlargement including the roots, the plexus and the proximal segments of the nerves). (2) CSA enlargement is typically more often seen in demyelinating neuropathies than in axonal types. Next to CSA enlargement of the whole nerve, some PNP reveal a fascicular enlargement pattern, in which one or more fascicles can be involved. In patients with multifocal motor neuropathy (MMN) single fascicles are enlarged next to normal sized fascicles (Figure 3D) [8,9]. Fascicle enlargement in PNP can also be regional (some fascicles enlarged in some nerves, sparing other nerves), differential (sparing some fascicles, but affecting all nerves) or generalized (affecting all fascicles and all nerves) (3). Depending on the number and size of affected fascicles, the CSA of the whole nerve can be enlarged as well. However, fascicle visualization and thus size interpretation must be handled with caution because the resolution and therefore the probe frequency plays an important role [8,10]. Recently, the focus has been further set on nerve echointensity [11,12,13,14]. The echo signal of the healthy nerve contains the hyperechoic area (i.e., the epineurium, the perineurium) and hypoechoic area (i.e., the nerve bundles). Padua and colleagues already described echointensity changes in some patients with chronic inflammatory demyelinating polyneuropathies (CIDP) [11]. Similar findings have been made by Haertig et al. and Fisse et al. [12,13]. Taken together, next to nerve enlargement, nerves can be hyperechoic (particularly fascicles due to scar tissue, fibrosis or axonal damage) or hypoechoic (maybe provoked by inflammation or edema). The qualitative description of the echo signal might be more suitable for daily routine; however, the quantitative analysis by software tools (grey scale analysis and fraction of black) is increasingly used as a more common method. (4) Concerning nerve vascularity, nerve elastography and the perinerval tissue, new insights have been published [15,16], but its role in PNP still remains unclear and thus description of these methods will not be discussed at this point.

## 3. General Aspects and Examination Protocols

So far, reference data for CSA in adults and children older than two years for most of the larger peripheral nerves have been published by many groups [17,18,19,20,21,22,23,24,25]. However, if values from literature are used, ethnic differences have to be kept in mind, i.e., CSA tend to be smaller in the Asian population [18,26]. The authors suggest obtaining your own reference values analogous to recommendations for nerve conduction studies. Reference data for nerve echointensity are sparse and only a few studies have published quantitative analysis values [14]. In daily clinical routine, a qualitative analysis in comparison to surrounding tissue, for example vessels, muscles, fat filled flat tunnels and fascia, should be used.

Another important point is to establish a scanning protocol, which includes the nerve and its measure points. Several analyzing protocols have been published so far [17,18,19,20,21,22,23,24,25,27]. Unfortunately, common protocols or guidelines (as in nerve conduction studies) do not exist so far [6]. Overall, analysis of all large nerves (>1 mm^2^) is feasible. We recommend a structured analysis of cervical roots (C5 and C6), vagus, median, ulnar and radial nerve, tibial, fibular and sural nerve and its measurement at predefined measurement points [20]. In cases of symmetric neuropathies, unilateral scans might be suitable, in cases of asymmetric neuropathy, bilateral scans are required. Each nerve should be scanned in its entire course as far as possible, whereas for reproducibility predefined landmarks should be used for measurement (see section scoring systems). To ensure exact data, the measurement of CSA and fascicle area should be carried out strictly within the hyperechoic rim and the echointensity analysis must be performed in predefined gains. The aim of this examination is to define neuropathies with enlarged nerves (hypertrophic neuropathies), and thus to visualize the nerve enlargement pattern as mentioned before. In the following sections we will describe known patterns of distinct neuropathies.

## 4. Hereditary Neuropathies

The most intriguing nerve enlargement can be found in patients with Charcot Marie Tooth (CMT) type 1 (CMT1) neuropathies, particularly type A [27,28,29,30,31,32,33,34,35,36,37,38]. Most of these patients (88–100%) exhibit a generalized nerve enlargement present at all sites with at least one region showing a CSA more than twice of the upper normal limit (Figure 2A,B). Nerve hypertrophy in CMT1 is already visible in children [27,33]. In CMT1B patients, nerves exhibit an enlarged CSA as well, but enlargement is less pronounced in comparison to CMT1A patients [37,38,39]. Other ultrasound findings in CMT1 types, for example CMT1C, have only rarely been reported regarding nerve size, but in most cases a similar pattern as in CMT1B has been found [32,37,38,39,40]. Recently, Castoro and colleagues described the case of a young patient with CMT4J, where a proximal predominant nerve enlargement pattern was seen [41].

In addition, the number of reported patients with a genetically confirmed neuropathy and a superimposed nerve inflammation increases rapidly [42]. In 1982, Peter J. Dyck and his colleagues were one of the first to describe a response to immune modulatory treatments in CMT patients [43]. In these overlap neuropathies a co-occurrence of a Guillian-Barre syndrome (GBS)- or CIDP-like disease course in patients with genetically confirmed polyneuropathies was reported. In all described cases, the onset of worsening occurred suddenly after a long period of a stable or slowly progressive disease course. Nerve enlargement seemed to decrease under immune modulatory therapy and symptoms improved, but further studies are needed to evaluate this entity. [44,45]

In contrast to demyelinating CMT1 types, in axonal CMT forms, CMT2, almost no nerve enlargement occurs. Merely, some fascicles can be enlarged, but CSA is broadly documented within reference ranges [30,34,36]. On the other hand, patients with X-linked CMT1 (CMT1X), show an intermediate pattern with moderate nerve enlargement, particularly in male patients [36].

Remarkably, the underlying genetic defect and not nerve conduction study (NCS) results (axonal or demyelinating) seems to determine the nerve morphology in CMT, considering that in neurofilament light polypeptide gene (NEFL) or myelin protein zero (MPZ) associated CMT1 or CMT2 similar ultrasound aspects are observed [32].

Finally, hereditary neuropathy with liability to pressure palsies (HNPP), mostly due to peripheral myelin protein 22 (PMP22) deletion, has a pathognomonic pattern of nerve enlargement at entrapment sites (e.g., carpal tunnel, cubital tunnel, fibular tunnel, and supinator loge) with normal nerve segment in between. Entrapment ratios (calculated by CSA at entrapment site divided by CSA at normal sites) are enlarged (e.g., wrist-to-forearm ratio), which enables differentiation to other inherited and demyelinating neuropathies [30,31,36,46,47,48,49,50]. Exceptions are sites with known long-lasting compression and consecutive tomaculae in the corresponding position. Straight anamnesis might elucidate the background of enlargement outside classical entrapment sites. Nevertheless, multifocal entrapment syndromes are not specific and can also occur in other neuropathies, like for example hereditary transthyretin related amyloidosis (ATTR) or diabetic neuropathy (DN) [5].

## 5. Hereditary Transthyretin Related and Sporadic Amyloidosis

ATTR is a systemic disorder leading to amyloid fibril deposition with cardiac dysfunction, carpal tunnel syndrome and severe autonomic and sensorimotor polyneuropathy [51]. Meanwhile, three drugs have been established complementing liver transplantation, the only therapy existing so far. These are TTR-stabilizer tafamidis, small interfering ribonucleic acid (RNA) patisiran and antisense oligonucleotide inotersen. As all of them are solely approved for first or second stages of ATTR and rareness of the disease often leads to delayed diagnosis, a good diagnostic tool for early and correct diagnosis is essential [52,53]. Magnetic Resonance (MR) neurography already proved its usefulness in detecting peripheral nerve pathology especially in lower limbs and even prior to the onset of symptoms in genetic variant carriers [2]. Further, this nerve enlargement can also be seen by ultrasound, particularly at entrapment sites (Figure 2C,D) [54]. Podnar et al. found that besides common entrapment sites, a pronounced nerve enlargement in a larger cohort of ATTR patients was also seen in proximal segments and plexus [55], and even of presymptomatic carriers [56]. This finding suggests ultrasound as an important tool to enable early treatment.

In contrast to ATTR, little is known about acquired amyloidosis. One case with amyloid light-chain (AL) amyloidosis is described with severe course and significant nerve enlargement suggesting a correlation between amyloid deposits in the nerves and mentioned enlargement; however, further studies are required [57].

## 6. Immune-Mediated Neuropathies

The role of imaging tools in CIDP, like MR neurography and HRUS, has been widely analyzed [3,11,12,13,58,59,60,61,62,63,64,65,66,67,68,69,70]. All authors describe nerve enlargement, particularly affecting proximal nerve segments and roots, which might facilitate a differentiation from CMT1 (Figure 3A,B). Enlargement can be found in up to 90% of patients (sensitivities range between 64 and 89% depending on the used scores and values), however to a very heterogeneous amount, ranging from a focal or regionally restricted to diffuse pattern. Even in patients with NCS not fulfilling the European Federation of Neurological Societies (EFNS) criteria, nerve enlargement can be observed with a high sensitivity, whereas the specificity of this finding is lower than this of NCS [71,72,73,74,75]. It is noteworthy that the amount of nerve enlargement does probably not correlate with disease severity, but with disease duration [59,64]. Interestingly, nerve enlargement is reversible in many cases, particularly in those with excellent therapeutic response [12,13,74].

Three distinct classes of nerve enlargement have been described [11,12]: Class 1 with hypoechoic nerve enlargement, Class 2 with hypo- and hyperechoic nerve enlargement and Class 3 with almost no nerve enlargement. Class 1 is the best treatment reactive, whereas Class 3 does respond least to therapy. Reasons might be a more chronic and axonal damage as well as fibrosis in Class 2 and 3. Further data will be needed to prove this suggestion. Additionally, ultrasound of the muscles might be useful as a prognostic marker to further evaluate axonal damage [76]. The role of ultrahigh-frequency ultrasound to detect fascicle affection in CIDP seems promising; however, it must be further proven [77] and the availability of these probes is restricted.

Only few data are available concerning imaging of paranodopathies, but the pattern seems to be similar to that seen in typical CIDP, probably more often affecting cranial nerves [78,79].

Only few reports concerning patients with Lewis-Sumner Syndrome (LSS) or multifocal acquired demyelinating sensory and motor neuropathy (MADSAM) are available [80,81,82,83,84,85,86]. Most authors mixed them with other CIDP types, which might hamper the right description of this immune-mediated subtype. Overall, patients with LSS show a similar pattern as those with CIDP, however with a more asymmetric distribution and sometimes enlargement in more distal segments [3]. In some cases, focal enlargement, restricted to one fascicle, can be seen and is correlated with nerve conduction block (Figure 3C) [81]. The enlargement however can be tremendous. Some patients even show enlargement of cranial nerves [84]. Scoring tools including the enlargement pattern, fascicle morphology and echotexture might help to differentiate several immune-mediated and hereditary neuropathies [87,88]. Just as suggested in CIDP, nerve enlargement in LSS might disappear or decrease under successful treatment [89,90].

In patients with monoclonal gammopathy of undetermined significance (MGUS) and polyneuropathy, we have to differentiate several subtypes of neuropathies [91]. This is most important as several subtypes implicate distinct therapies [92]. Most patients exhibit an axonal NCS type with distal symmetric polyneuropathy and no nerve enlargement, probably with no immune-mediated pathology [93]. These consequently do not respond well to immunomodulatory treatment. However, if nerve enlargement is found despite axonal neuropathy type in MGUS, immunotherapy might be an option, as shown in two cases of M-protein related neuropathies [93]. Furthermore, we can find CIDP-like types in all immunoglobulin-mediated variants (MGUS-CIDP). Their pattern appears the same as in classical CIDP. Importantly, in immunoglobulin M (IgM)–MGUS, patients with positive anti-myelin associated glycoprotein (MAG) antibodies show a poor response to treatment with steroids and intravenous immunoglobulins (IVIG). Here, anti-CD20 antibodies or tyrosine kinase inhibitors seem to be more effective. In anti-MAG neuropathy, nerve enlargement pattern can be similar to that in LSS and CIDP, for example also affecting the roots, while NCS data exhibit a distal predominant pattern, but can also just be mild [93,94]. Furthermore, small sensory distal branches can be affected [95].

So far, less is known about POEMS (polyneuropathy, organomegaly, endocrinopathy, monoclonal protein, skin changes) patients, who exhibit demyelinating NCS with uniform pattern and, as described so far, enlarged CSA especially in entrapment sites or slightly enlarged nerves as compared to healthy controls [96,97]. This enlargement might decrease with successful treatment.

Another immune-mediated neuropathy affecting several pure motor nerves in one or more limbs with mostly asymmetric onset is the multifocal motor neuropathy (MMN [98]. In NCS, conduction blocks are the main finding, however in very proximal segments this examination might be difficult [99]. Often present in this case are GM1-IgM antibodies with sensitivity of up to 70%, but low specificity [100]. Treatment with immunoglobulins is the only one effective so far, thus, early differentiation from clinically similar diseases like amyotrophic lateral sclerosis (ALS), inclusion body myositis or pure motor CIDP is crucial. Nerve enlargement was first described by Beekman et al. in MMN [101], followed by several other authors [9,102,103,104,105]. In MMN, nerve enlargement is much more regionally or focally limited than in LSS and CIDP, often only affecting one or few fascicles (Figure 3D). Herein, one can often detect electrophysiological conduction blocks, but there is not always a correlation to fascicular enlargement [9]. Fascicle enlargement can disappear with treatment with intravenous or subcutaneous immunoglobulins. Distribution is mostly asymmetric, showing side-to-side differences and greater intra-nerve variability than CIDP patients [106]. Sensory nerves are spared in MMN, in NCS as well as in ultrasound [102]. By finding multifocal nerve enlargement, differentiation from ALS might be facilitated. However, slight nerve enlargement might also be seen in the beginning stage and probably in the inflammatory type of ALS [107], even though in contrast, MMN nerves in ALS develop atrophy during the course of disease [108]. According to literature, sensitivity and specificity of ultrasound ranges between 88–100% and 93–100%.

The acute variant of an immune-mediated neuropathy, GBS, is mostly preceded by an infectious gastrointestinal or pulmonary disease, most frequently by Campylobacter jejuni infection. Other associations with Zika, Cytomegaly and severe acute respiratory syndrome coronavirus type 2 (SARS-CoV-2) infection have been described [109,110,111]. Clinical symptoms are distally arising sensory and predominantly motor symptoms leading to tetraparesis including autonomic dysregulation and cranial nerve palsies. Several clinical variants and atypical cases exist. CSF typically exhibits cytalbuminic dissociation, however often not before week two after clinical onset. NCS data can be inevident in the first days, but are later observed on proximal pathologies or multifocal A-waves. Most patients exhibit prominent nerve root and vagus nerve enlargement in ultrasound already in the first days of symptoms [112,113,114]. Sensitivity, specificity, and positive predictive values are >85% in literature. Root enlargement resembles radiculitis in GBS, distal segments are not predominantly swollen. Vagus nerve enlargement seems to correlate with autonomic dysregulation and thus might promote the risk of stratification in the future [112]. These findings can occur in axonal and demyelinating types [114,115,116]. Recently, nerve enlargement has also been described in Post-SARS-CoV-2-GBS [117]. Importantly, nerve root enlargement disappears or might decrease in most cases, equally in adults and in children, after successful treatment within three months. Early decrease of nerve enlargement in GBS under therapy is opposed to patients with CIDP, which facilitates differentiation of both entities [118,119,120]. In the beginning a differentiation of GBS and acute-onset CIDP might be possible by the more prominent swelling of peripheral sensorimotor nerves in the latter case. Further sensory nerve swelling also seems to be pathognomonic for chronic immune-mediated neuropathies [120].

The Miller Fisher syndrome (MFS), presenting with ataxia, areflexia and ophthalmoplegia, is a variant of GBS with antiGQ1b IgG antibodies, often presenting as an overlap syndrome with MFS and GBS symptoms [109], also showing enlargement of the roots and cranial nerves [116].

## 7. Diabetic Neuropathies

Although DN are a large and important part of all neuropathies worldwide, literature concerning ultrasound in this entity is not rich. Some authors suggest that DN does not cause nerve enlargement, particularly the classical distal-symmetric axonal type [5,121]. However, this is consistent with the finding that we see nerve enlargement in demyelinating neuropathies rather than in the axonal forms; still, even in the mixed axonal and demyelinating forms, there was no difference between DN and controls [121]. Exceptions are the entrapment areae, which often exhibit nerve enlargement (e.g., tarsal tunnel, carpal tunnel) [122]. Still, some other authors found that nerve size might increase in DN to some amount compared to controls, depending on severity and in one study even correlating with Hemoglobin A1c (HbA1c) independent of the presence of a neuropathy [122,123,124,125,126,127]. Still, with exact regard on the published data, nerve enlargement in DN does not exceed upper reference values. In contrast, the vagus nerve was found to be smaller and atrophic in patients with diabetes [128], maybe explaining autonomic dysregulation in DN. The influence of several subtypes and its distinct NCS pattern (e.g., axonal or demyelinating), as well as therapeutic approaches are so far missing in ultrasound studies. Thus, interpretation of ultrasound findings in diabetes patients has to be done with care. Significant and multifocal nerve enlargement outside of entrapment sites in a patient with diabetes must raise attention for a second reason for neuropathy, for example inflammation or underlying hereditary neuropathy. Further, there have been recent descriptions of increased nerve stiffness, as evaluated by shear wave elastography [16]; however, the method has to be confirmed in larger studies. With regard to its importance, further studies clearly differentiating types of diabetes, therapeutic concepts and neuropathy forms are required to the authors´ opinion.

## 8. Other Axonal Neuropathies

Until now, ultrasound patterns for axonal neuropathies have mostly been described as for subgroups of large cohorts, not differentiating between toxic, metabolic, idiopathic or inherited axonal neuropathies [58,129,130]. Overall spoken, nerve enlargement is not existent in most axonal neuropathies. Exceptions in acquired immune-mediated types have been described, such as sarcoidosis [129], although here nerve enlargement did not correlate with clinical function or electrophysiological findings. Furthermore, similar results were found for vasculitic neuropathies [130,131,132,133] as well as infectious neuritis (e.g., borreliosis, hepatitis E [134,135]), in which focal nerve enlargement might be seen. Besides the exceptions mentioned above, nerve enlargement is particularly seen in clinically affected nerves. Notably, distal sensory segments can be significantly involved, which might serve as a localization for further diagnostics, like for example nerve biopsy [132]. Sonographic differentiation from demyelinating immune-mediated neuropathies is possible as proximal parts such as roots and plexus are mostly spared in vasculitis and enlargement is only slight to moderate with restricted expansion [136]. Data regarding nerve alterations in chemotherapy induced neuropathies are inconsistent; however, most authors state no or only focal nerve enlargement in those patients. In patients with taxane treatment though, smaller CSA values of the sural nerve were found [137], whereas in oxaliplatine treated patients a slight increase of the tibial and peroneal nerve was reported [138]. This difference might be due to the distinct pathology (neuropathy vs. neuronopathy), still, larger studies are mandatory to confirm these data. Neurodegenerative axonal neuropathies might exhibit small nerve CSA, as shown for progressive ataxia syndromes with neuropathy such as Cerebellar ataxia neuropathy vestibular areflexia syndrome (CANVAS) and spinocerebellar ataxia type 2 [139].

Another entity of neuropathies, which has recently been more and more diagnosed and though is not entirely understood, is the small fiber neuropathy (SFN). SFN often presents with neuropathic pain and numbness while revealing normal NCS. The use of nerve ultrasound in SFN is currently under investigation.

## 9. Neurolymphomatosis and GRAFT Versus Host Diseases

Neoplastic affection of the peripheral nerve system (PNS) in lymphoma is a rare manifestation called neurolymphomatosis [140] and arises as mono- or multiplex polyneuropathy. Tremendous nerve enlargement seen by ultrasound might help to detect this pathology, which finally must be confirmed by biopsy or positron emission tomography (PET)-imaging [141].

Graft versus host disease (GvHD) after allogenic marrow transplantation mostly affects the skin and the gastrointestinal system. However, neuromuscular involvement can be an issue [142]. If the PNS is involved, courses exhibit GBS- or CIDP-like features. NCS data and, if demyelination is found, nerve ultrasound can help to diagnose GvHD neuropathy. However, diagnosis might also be hampered by preexisting toxic axonal neuropathy due to chemotherapy. Here, nerve enlargement, comparable to CIDP or GBS findings, might help to identify PNS-GvHD [143,144]. Treatment options are comparable to those used for the autoimmune mediated neuropathies but must also be in concordance with treatment concerning the transplant.

## 10. Storage Diseases

**Figure 4:**
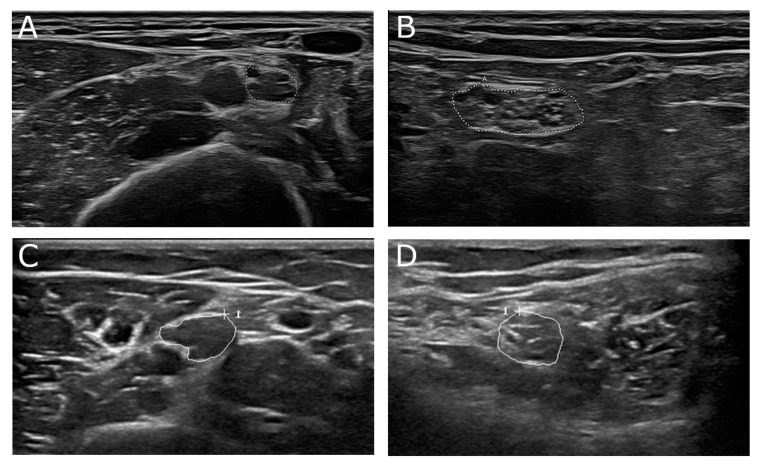
(**A**,**B**) 68-year-old male patient with genetically proven adrenomyeloneuropathy, an adult form of adrenoleukodystrophy. Nerve conduction studies showed a demyelinating damage pattern. In nerve ultrasound focal nerve enlargement was detected with a UPSS of 7 points. (**A**) Median nerve at upper arm segment (cross-sectional area (CSA) 15 mm^2^, normal value 12 mm^2^). (**B**) Tibial nerve at popliteal fossa (CSA 51 mm^2^, normal value 33 mm^2^). (**C**,**D**) 16-year-old female patient with juvenile form of metachromatic leukodystrophy. Focal nerve enlargement was seen in median nerve (**C**, CSA 20 mm^2^) at proximal nerve sites next to normal nerve segments in other nerves, i.e., tibial nerve (**D**, CSA 23 mm^2^). Areae enclosed by the discontinuous points in picture A and B depict CSA of the described nerve section, “A”means “area”. Areae enclosed by circles in pictures C and D depict CSA of the described nerve section, “+” shows curser position, “1” the count of measured areae.

Growing data are available concerning rare storage diseases, for example metachromatic leucodystrophies, adrenoleucodystrophies, xantochromatosis or glucocerebrosidosis [27,145,146,147]. Until now, even though only limited data exist, most of the patients with storage diseases show nerve enlargement, possibly due to accumulation of intermediate metabolites within the myelin sheets, according to demyelinating NCS in most of these cases. Two examples of enlarged nerves in these rare neuropathies are given in Figure 4. If patients with central nervous deficits and polyneuropathy show coexisting nerve enlargement in nerve ultrasound, testing for rare lysosomal storage diseases should be initiated.

## 11. Motor neuron Diseases

Next to muscle ultrasound in motor neuron diseases such as ALS, nerve ultrasound can contribute important information concerning the nervous system [108,148,149]. Particularly atrophy of the distal ulnar nerve or the roots [104,108,150], for example, in lower motor neuron variants of ALS, might be a prognostic marker. Interestingly, this could not be shown in primary lateral sclerosis (PLS). Findings are thus indicating ultrasound as the key tool to identify PLS from the upper motor neuron dominant variant of ALS, which is clinically difficult to separate [151]. In ALS, nerve atrophy seems to progress during disease [104,148,151,152]. However, in the early course of disease, even focal nerve enlargement can rarely be seen [107]. Still, clinical presentation and course remains the most important fact. Nevertheless, if nerve enlargement is seen in patients with severe NCS pathology with or without conduction blocks [102,103], a possible inflammation must be included in the therapeutic concept.

## 12. Scoring Systems

In general, demyelinating neuropathies regularly show nerve enlargement, whereas axonal ones do not [34,129,130]. However, many exceptions exist such as vasculitic PNP or amyloidosis. Therefore, a quantification of nerve enlargement including pattern analysis, anatomical predominance, echointensity and amount of nerve enlargement is necessary to avoid mis- or overinterpretation. Some authors suggest scoring tools to better decrypt several neuropathies by summing up characteristics of nerve alterations [149]. Craig Zaidman was the first to develop a classification of normal sized nerves, mild, regional or diffuse nerve enlargement for the differentiation of axonal neuropathies, GBS, CIDP and CMT1A [34]. CMT1A patients mostly showed a diffuse pattern, whereas CIDP patients exhibited a regional pattern.

**Figure 5:**
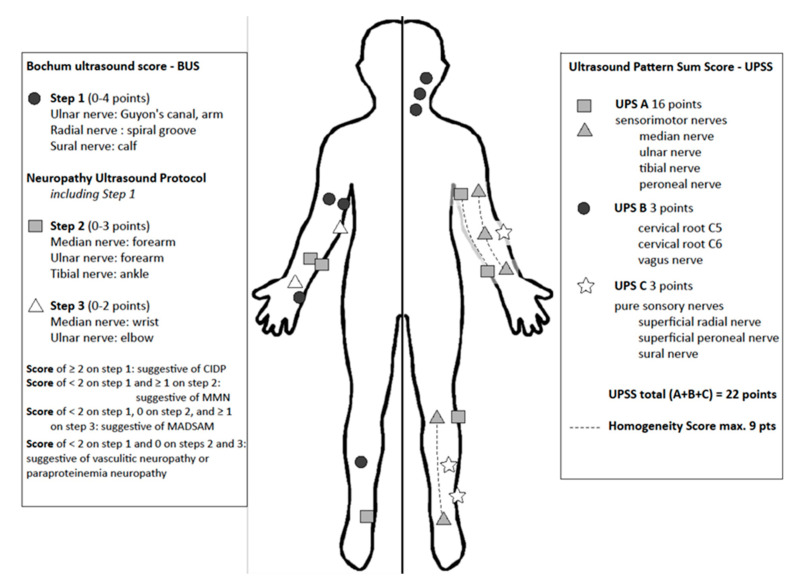
Measure points and scoring system of Bochum Ultrasound Score (BUS, left side), Neuropathy Ultrasound Protocol (NUP) and Ultrasound Pattern Sum Score (UPSS, right side). BUS and NUP are applied in three steps according to number of enlarged nerves. UPSS is performed in one step and the addition of homogeneity score allows differentiation of nerve in homogenous or heterogenous enlargement patterns.

Our colleagues from Bochum University Hospital developed the Bochum Ultrasound Score (BUS), which enables differentiation between GBS and CIDP in the early disease course [150,153]. More than two enlarged nerves in an analysis of the ulnar nerve (examined at Guyon loge and upper arm), the radial nerve (spiral groove) and the sural nerve points to the diagnosis of CIDP (Figure 5). This score is a reliable fast-track tool, which can be easily used in each neurophysiology unit.

A further established score is the ultrasound pattern sum score (UPSS) [19,87,138], which quantifies nerve enlargement at several nerve segments. It is subdivided in three parts: UPS *A* for peripheral sensorimotor nerves, UPS *B* for roots C5 and 6 and vagus nerve and UPS *C* for sensory nerves. Additionally, it includes further evaluation of homogeneity of nerve enlargement [87]. In this score, a maximum of 22 points can be reached, resembling significant nerve enlargement in all nerve segments with homogeneous distribution, as for example found in CMT1A. On the other hand, an enlarged UPSB score next to normal UPSA and UPSC scores hints to the diagnosis of GBS in a typical clinical context or any other pure radiculitis. An overall score lower than three points excludes inflammation or heredity of neuropathy with a negative predictive value of more than 90%. A UPS A score larger than three points without root enlargement (UPS B) suggests the diagnosis of vasculitis. Additional evaluation of homogeneity of the nerves (median, ulnar and tibial nerve) enables differentiation of CMT1 and CIDP [87].

Next to the UPSS or BUS, Padua et al. described several echointensity classes in immune-mediated neuropathies [11], which proved its strength in a prospective study of Haertig et al. [12]. Further, regional or differential fascicle enlargement might be a hallmark in MMN or LSS [8,9]. Thus, a combination of nerve enlargement quantification, fascicle evaluation, echointensity classification and entrapment analysis might contribute to a better distinction of neuropathies. However, multicenter evaluation is still required concerning all mentioned scoring tools.

A summary of BUS and UPSS is shown in Figure 5. Noteworthy for these scores is their character as additive tools. These scoring tools must be interpreted in the context of nerve conduction, clinical findings and medical history. A UPSS Score of zero points does not exclude immune-mediated forms, for example GBS. However, the accuracy of the Zaidman classification, the UPSS and the BUS was rather excellent in a retrospective meta-analysis so far [149].

## 13. Practical Approach Combining Electrophysiology and Nerve Ultrasound

**Figure 6:**
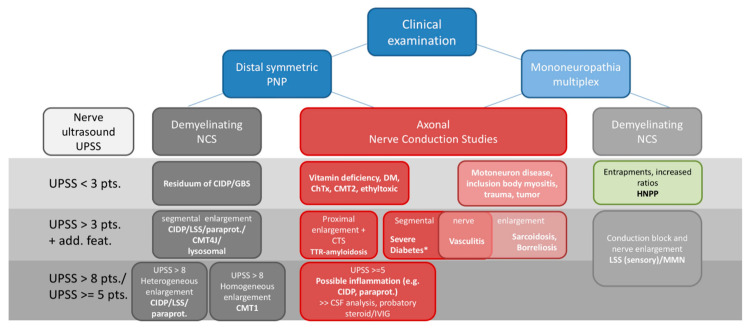
Proposed diagnostic algorithm and possible, most common differential diagnosis for clinical routine including clinical examination, nerve conduction studies and nerve ultrasound. * an additional inflammatory component might play a role in nerve enlargement. Abbreviations: ChTx = chemotherapy, CIDP = chronic inflammatory demyelinating polyneuropathy, CMT = Charcot Marie Tooth, CSF = cerebrospinal fluid analysis, CTS = carpal tunnel syndrome, DM = diabetes mellitus, HNPP = hereditary neuropathy with pressure palsies, GBS = Guillain-Barre-Syndrome, IVIG = intravenous immunoglobulin, LSS = Lewis-Sumner Syndrome, MMN = multifocal motor neuropathy, NCS = nerve conduction studies, paraprot. = paraproteinemic polyneuropathies, PNP = polyneuropathies, TTR = hereditary transthyretin related amyloidosis, UPSS = ultrasound pattern sum score.

Although many aspects concerning the value of nerve ultrasound are still missing in the diagnosis of PNP, its use for several neuropathies has been widely proven. It might help to reduce the high number of unknown etiologies and thus support practitioners to target their diagnostic steps. Still, before performing ultrasound, profound knowledge of ultrasound technique, anatomy of the musculoskeletal and nervous system as well as its distinct pathologies is essential [6,7]. Thus, experts recommend international trainings and guidelines to learn and to perform ultrasound with common protocols [154,155]. Distinct ultrasound devices and probes might hamper this operationalization; however, this hurdle was already taken by other disciplines, too. Nevertheless, the authors propose the additive use of ultrasound, particularly in unclear cases of suggested inflammation. Further, its use as a biomarker for therapeutic response might be suitable in daily routine. Ultrasound is fast and cost-effective and thus could be widely used in contrast to other imaging tools [156]. Finally, we recommend an algorithm for how to handle ultrasound as an additive tool in the context of clinical examination and nerve conduction studies concerning the most important PNP variants and mimics (Figure 6).

## 14. Limitations and Conclusions

Examiners using ultrasound must bear in mind the limitations of nerve ultrasound. Most of the studies are single center based and thus sensitivities and specificities must be interpreted with care. Further, many distinct scoring tools have been used, which influenced the sensitivity and specificity significantly. To get profound values, multicentric studies including several neuropathy types are mandatory in the future. Normal data might depend on ethnicity, weight, height, and sex [17,18,19,20]. Particularly the examiner experience and the used ultrasound tool might influence image quality and thus interpretation. Profound knowledge of ultrasound is highly recommended and crucial for an accurate diagnosis. Clinical examination and NCS remain the actual gold standard for the evaluation of the peripheral nerve system and are indispensable. Of note, ultrasound has its limitations in deep layers, for example, lumbar plexus, lumbar roots and deep smaller nerves. Further, little is known about contrast enhanced nerve ultrasound. Herein, MRI is superior to ultrasound and thus the combination of both tools can be necessary in some neuropathies. Nonetheless, ultrasound might help to detect nerve alterations in otherwise non classifiable neuropathies (e.g., axonal predominant inflammatory neuropathy [12,72], or to differentiate between inherited and acquired neuropathies. Further, ultrasound might serve as a biomarker for therapy control; however, multicentric data are needed for this indication [12]. Table 1 summarizes all important key features concerning several polyneuropathies.

Therefore, ultrasound should be included as a basic tool in every neurophysiology department as it adds important information.

## Figures and Tables

**Table 1:** Overview of typical nerve conduction and ultrasound findings in various forms of polyneuropathies. A detailed description including references can be found in the text in the respective text sections.

Diagnosis	Predominant Nerve Conduction Findings	Common Ultrasound Findings	Echointensity	Scores/Cut-Offs
**Hereditary Neuropathies**				
CMT1a	Demyelinating	Homogeneous nerve enlargement	Reduced	UPSS > 8 HS ≥ 4
CMT1b	Demyelinating	Homogeneous nerve enlargement (smaller than CMT1a)	Reduced	UPSS > 8 HS > 3
Other CMT1/4	Demyelinating	Regional nerve enlargement (heterogeneous)	*Insufficient data*	*Insufficient data*
CMT2	Axonal	No significant nerve enlargement	*Insufficient data*	UPSS < 3
HNPP	Nerve entrapment	Enlargement at entrapment sites	Reduced at entrapment sites	UPSS < 3 Entrapment ratios > 1,5
ATTR	Axonal and nerve entrapment	Proximal nerve enlargement and at entrapment sites	Reduced	UPSS > 3, entrapment
SCA neuropathy/ CANVAS	Axonal (sensory dominant)	No nerve enlargement (upper limb nerves <5mm^2^ in CANVAS patients)	Increased	Upper limb nerves <5mm^2^
**Storage diseases**				
Metachromatic and Adrenoleucodystrophies/ Cerebrotendinous Xanthochromatosis	Demyelinating, homogeneous	Diffuse nerve enlargement	Reduced	UPSS > 3 (AMN)
**Acquired neuropathies**				
GBS	Axonal (AMAN/AMSAN), demyelinating (AIDP/MFS)	Nerve root and vagus nerve enlargement	Reduced	UPSS < 5 UPSB ≥ 1 UPSC < 1 BUS < 2
CIDP	Demyelinating EFNS criteria (symmetric)	Heterogeneous enlargement of all nerves (proximal predominant, median nerve, ulnar nerve, nerve roots and plexus)	Heterogeneous (Class 1 reduced, Class 2 increased, Class 3 mixed/ not enlarged)	UPSS > 5 BUS > 1
MADSAM	Demyelinating with temporal dispersion and conduction block (asymmetric)	Fascicular and regionally restricted nerve enlargement, also resembling CIDP pattern	Similar to CIDP	UPSS > 3 UPSC > 0
Multifocal Motor Neuropathy	Conduction block, only motor nerves affected	Fascicular and regionally restricted nerve enlargement, correlating with conduction block in up to 75%	Reduced> increased	UPSS > 3 UPSC < 1 (no sensory nerves affected)
Anti-MAG PNP	Demyelinating	Similar to CIDP	Similar to CIDP	Similar to CIDP
MGUS-CIDP	Similar to CIDP	Similar to CIDP	Similar to CIDP	Similar to CIDP
Other PNP associated with MGUS	Axonal	No enlargement	*Insufficient data*	UPSS < 3
Multiple Myeloma associated PNP	Axonal	No enlargement	*Insufficient data*	UPSS < 3
POEMS	Demyelinating (homogeneous)	Heterogeneous nerve enlargement, entrapment sites	*Insufficient data*	UPSS > 3
Vasculitis	Axonal (asymmetric)	Focal nerve enlargement in symptomatic nerves	Increased	UPSS 3–9 Multifocal nerve enlargement of ≥1 nerve
Diabetic PNP	Axonal > >demyelinating	Slight nerve enlargement (e.g., entrapment sites)	Inconsistent data	UPSS < 3
ChTx induced neuropathies	Axonal	No significant nerve enlargement	Increased	UPSS < 3
Other axonal neuropathies (e.g., vitamin deficiency)	Axonal	No nerve enlargement	*Insufficient data*	UPSS < 3

Abbreviations: AMAN: Acute motor axonal neuropathy, AMSAN: Acute motor-sensory axonal neuropathy, AIDP: Acute inflammatory demyelinating polyneuropathy, AMN: Adrenoleucodystrophies, ATTR: Hereditary transthyretin related amyloidosis, BUS: Bochum Ultrasound Score, CANVAS: Cerebellar ataxia neuropathy vestibular areflexia syndrome, ChTx: Chemotherapy, CIDP: Chronic inflammatory demyelinating polyneuropathies, CMT: Charcot Marie Tooth Neuropathy, GBS: Guillain-Barre syndrome, HNPP: Hereditary neuropathy with liability to pressure palsies, HS: Homogeneity score, MADSAM: Multifocal acquired demyelinating sensory and motor neuropathy, MAG: Myelin associated glycoprotein, MFS: Miller-Fisher syndrome, MGUS: Monoclonal gammopathy of undetermined significance, PNP: Polyneuropathy, POEMS: Polyneuropathy, organomegaly, endocrinopathy, monoclonal protein, skin changes, SCA: Spino-cerebellar ataxia, UPSA-C: Ultrasound pattern score A-C, UPSS: Ultrasound pattern sum score.

## Abbreviations

AL	Amyloid light-chain
ALS	Amyotrophic lateral sclerosis
ATTR	Hereditary transthyretin related amyloidosis
BUS	Bochum Ultrasound Score
CANVAS	Cerebellar ataxia neuropathy vestibular areflexia syndrome
CIDP	Chronic inflammatory demyelinating polyneuropathies
CMT	Charcot Marie Tooth
CMT1X	X-linked Charcot Marie Tooth type 1
CSA	Cross-sectional area
CSF	Cerebrospinal fluid
DN	Diabetic neuropathy
EFNS	European Federation of Neurological Societies
GBS	Guillain-Barre syndrome
GvHD	Graft versus host disease
HbA1c	Hemoglobin A1c
HNPP	Hereditary neuropathy with liability to pressure palsies
HRUS	High-resolution nerve ultrasound
IgM	Immunoglobulin M
IVIG	Intravenous immunoglobulins
LSS	Lewis-Sumner Syndrome
MADSAM	Multifocal acquired demyelinating sensory and motor neuropathy
MAG	Myelin associated glycoprotein
MFS	Miller Fisher syndrome
MGUS	Monoclonal gammopathy of undetermined significance
MMN	Multifocal motor neuropathy
MPZ	Myelin protein zero
MR	Magnetic Resonance
MRI	Magnetic Resonance Imaging
NCS	Nerve conduction study
NEFL	Neurofilament light polypeptide gene
NUP	Neuropathy Ultrasound Protocol
PET	Positron emission tomography
PLS	Primary lateral sclerosis
PMP22	Peripheral myelin protein 22
PNP	Polyneuropathies
PNS	Peripheral nerve system
POEMS	polyneuropathy, organomegaly, endocrinopathy, monoclonal protein, skin changes
RNA	Ribonucleic acid
SARS-CoV-2	Severe acute respiratory syndrome coronavirus type 2
SFN	Small fiber neuropathy
TTR	Hereditary Transthyretin
UPSS	Ultrasound pattern sum score
